# Factors Influencing the Use of Patient-Specific Instrumentation (PSI) in Shoulder Arthroplasty: A Single-Centre Study

**DOI:** 10.7759/cureus.100208

**Published:** 2025-12-27

**Authors:** Srikiran Thalanki, Adam Daneshyar, Anurag Dixit, Omar Mostafa, Adrian William Simons, Tim McBride

**Affiliations:** 1 Trauma and Orthopaedics, The Royal Wolverhampton NHS Trust, Wolverhampton, GBR; 2 Trauma and Orthopaedics, Russells Hall Hospital, Birmingham, GBR; 3 Trauma and Orthopaedics, Cardiff and Vale University Health Board, Cardiff, GBR; 4 Trauma and Orthopaedics, Birmingham Orthopaedic Training Programme, Birmingham, GBR

**Keywords:** computer assisted instrument guidance, customized treatment plan, glenoid morphology, glenoid version, patient specific implant (psi), pre-operative planning, reverse shoulder arthroplasty, shoulder arthroplasty/replacement

## Abstract

Background

Accurate component placement is vital for shoulder arthroplasty success, with poor positioning linked to a range of clinical complications. This study aims to identify which patient, anatomical and surgical factors influenced the adoption of patient-specific instrumentation (PSI) for glenoid component placement. These included patient demographics, implant type, glenoid version and inclination, glenoid dimensions, use of augmentation, and surgeon-related factors.

Methodology

A retrospective decision-analysis study of 191 shoulder arthroplasty cases (reverse and anatomical) conducted between 2021 and 2024 at a single NHS trust. Preoperative imaging protocols, component planning, degree of deformity and intraoperative decision-making were examined. The primary outcome was the association between anatomical factors and PSI use. Statistical analysis performed using SPSS v16 (SPSS Inc., Chicago, IL).

Results

PSI use was strongly associated with increased glenoid retroversion (Cramér’s V = 0.37, *P* < 0.001) and superior inclination (Cramér’s V = 0.30, *P* = 0.001), and moderately associated with the use of medium and large glenoid augments (Cramér’s V = 0.33, *P* < 0.001). No significant association was observed between PSI use and patient age, sex, surgical side, glenoid anteroposterior width, or vault depth (all Cramér’s V < 0.15).

Conclusions

PSI is primarily adopted in cases with complex glenoid deformities. This study analyses decision-making patterns rather than clinical effectiveness. This may help inform future work towards more consistent PSI selection.

## Introduction

Accurate component placement is crucial for the success of total shoulder arthroplasty (TSA). There is strong evidence linking glenoid malposition to issues of loosening, instability and component failure [1-3]. Achieving optimal glenoid positioning intraoperatively is influenced by glenoid exposure, complex pathoanatomic factors, the surgeon’s experience and instrument limitations.

For reverse TSA (rTSA), the literature recommends glenoid baseplate placement with 5°-10° of inferior inclination and approximately 10° of retroversion to lowers risk of notching and optimise stability. Conversely, anatomical TSA (aTSA) aims for 0° inclination and neutral version. However, executing these targets intraoperatively is challenging and complicated by anatomical deformity, limited exposure, and conventional jig constraints. The use of patient-specific instrumentation (PSI) in arthritic shoulders is more accurate than standard instrumentation in both inclination and version measurements across both aTSA and rTSA procedures [4].

The emergence of three-dimensional (3D) computed tomography (CT) imaging has facilitated accurate preoperative assessment and planning. These scans allow for detailed visualisation and virtual simulation of PSI component placement using planning software, and allow for more accurate and guided instrument insertion during surgery [5].

PSI also offers several other advantages, including reduced neurovascular injury in the spinoglenoid notch [6]. The use of PSI with 3D preoperative planning led to the use of fewer screws, longer screws and screws placed with minimal deviation [7].

PSI is a rapidly growing technology with a strong value proposition, particularly for complex cases, yet the deciding factors for its adoption remain poorly defined in current literature.

This study aimed to analyse patient-, anatomical-, and operative-related factors influencing the decision to use PSI in shoulder arthroplasty. Clinical and functional outcomes were not evaluated.

## Materials and methods

This is a retrospective cohort study of all primary shoulder arthroplasty procedures performed by two consultants at The Royal Wolverhampton NHS Trust from 2021 to 2024. Ethical approval was not required, as this retrospective analysis did not affect patient care. The study was registered on the local audit database.

Selection 

Our inclusion criteria involved adult patients undergoing primary shoulder arthroplasty (reverse or anatomical) with complete preoperative CT imaging, planned according to Zimmer Biomet protocol and using Signature One software [8]. We excluded revision cases, incomplete imaging or missing data.

Peri-operative planning

Preoperative CT scans were performed for all cases following the Zimmer Biomet protocol, allowing detailed assessment of glenoid morphology, including retroversion, inclination, AP diameter, and vault depth. The consultant surgeon reviewed the CT data using virtual planning software and decided whether to use PSI or conventional jigs during the preoperative planning phase. All surgeries were performed through a standard deltopectoral approach.

Data collection and statistical analysis 

Patient demographics, anatomical parameters, augment use and PSI selection were systematically recorded. Statistical analysis employed the Shapiro-Wilk test for data normality and chi-square/regression analysis for group-wise comparisons (SPSS v16, SPSS Inc., Chicago, IL). Significance was set at *P *< 0.05.

## Results

Out of 191 shoulder arthroplasty cases included in the study, 116 (60.7%) were performed using PSI. PSI was more frequently utilised in rTSA cases (101/156, 64.7%) compared with anatomical TSA cases (15/35, 42.9%), and this difference was statistically significant (χ²(1) = 4.86, *P *= 0.027, Cramér’s V = 0.16) (Table 1).

**Table 1:** Demographic and surgical factors affecting PSI use. PSI, patient-specific instrumentation

Variable	χ²	df	*P*-value	Cramér’s V	Interpretation
Age category	3.57	2	0.168	0.14	No significant difference
Gender	0.24	1	0.516	0.04	No significant difference
Laterality	2.46	1	0.086	0.11	No significant difference
Surgeon	3.32	1	0.049	0.13	Significant surgeon preference

Demographic characteristics were then examined to determine their association with PSI use. PSI utilisation did not vary significantly across age categories (χ²(2) = 3.57, *P *= 0.168, Cramér’s V = 0.14), gender (χ²(1) = 0.24, *P* = 0.516, Cramér’s V = 0.04), or side of surgery (χ²(1) = 2.46, *P* = 0.086, Cramér’s V = 0.11). However, surgeon preference demonstrated a borderline significant association, with one surgeon using PSI more frequently (χ²(1) = 3.32, *P* = 0.049, Cramér’s V = 0.13) (Table 1).

Examination of anatomical and radiographic parameters demonstrated multiple statistically significant associations with PSI utilisation. Glenoid retroversion was strongly associated with PSI use (χ²(3) = 26.04, *P *< 0.001, Cramér’s V = 0.37). Median retroversion was higher in the PSI group (9.5° vs. 4.5°), and PSI was adopted in 23 of 26 cases (88.5%) with retroversion greater than 15°. Superior inclination also showed a significant association (χ²(3) = 17.51, *P* = 0001, Cramér’s V = 0.30), with PSI being used in nearly 90% of cases where inclination exceeded 10° (Table 2).

**Table 2:** Anatomical and intraoperative factors affecting PSI use. PSI, patient-specific instrumentation; RV, retroversion; SI, superior inclination; TSA, total shoulder arthroplasty; rTSA, reverse total shoulder arthroplasty

Variable	χ²	df	*P*-value	Cramér’s V	Interpretation
Glenoid retroversion	26.04	3	<0.001	0.37	Strong association - PSI used more with higher RV
Superior inclination	17.51	3	0.001	0.3	Moderate association - PSI used more with higher SI
Glenoid augment	20.99	3	<0.001	0.33	Moderate association - Use of medium/large augments tends to PSI use
Implant type (TSA vs. rTSA)	4.86	1	0.027	0.16	Small-moderate association
AP width	0.39	2	0.824	0.04	No association
Vault depth	2.53	2	0.282	0.12	No association
Central screw length	0.61	2	0.735	0.07	No association

Similarly, the need for glenoid augmentation influenced implant strategy. Patients requiring medium or large glenoid augments demonstrated higher use of PSI (χ²(3) = 20.99, *P* < 0.001, Cramér’s V = 0.33), with 40/46 (86.9%) large-augment cases managed using PSI. No significant association was observed between PSI use and AP width (χ²(2) = 0.39, *P* = 0.824, Cramér’s V = 0.04) or vault depth (χ²(2) = 2.53, *P *= 0.282, Cramér’s V = 0.12), with both dimensions being distributed similarly between groups (Table 2).

Central screw length also demonstrated no statistically significant difference between groups (χ²(2) = 0.61, *P *= 0.735, Cramér’s V = 0.07). Although PSI cases had a slightly greater median screw length (35 mm vs. 30 mm), the difference was not clinically meaningful, and screw length distribution did not influence PSI utilisation (Table 2).

**Figure 1:**
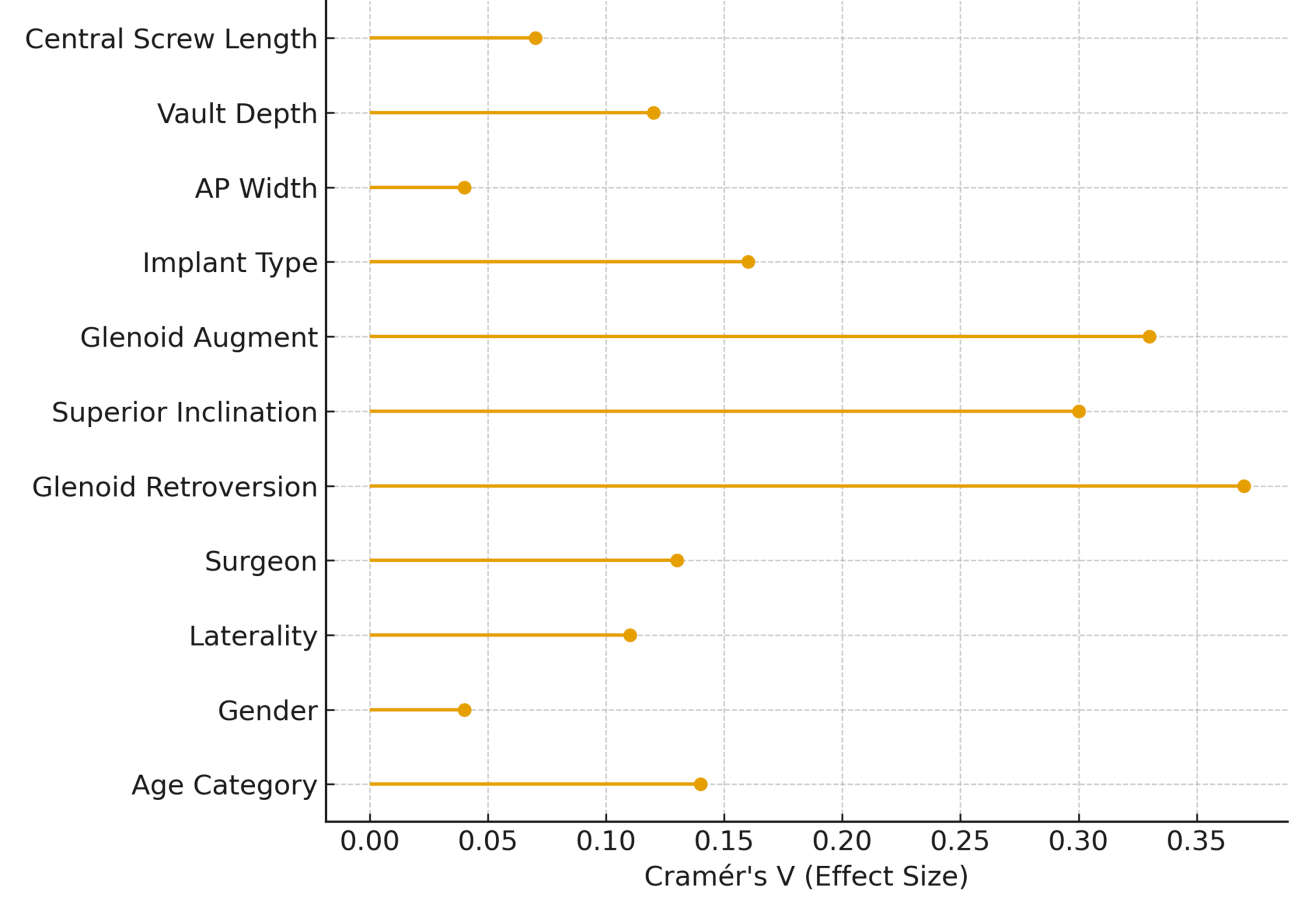
Forest plot of effect sizes for PSI use predictors. Each line in the plot shows how strongly each variable is linked to PSI use in shoulder arthroplasty. The longer the line, the stronger the association (measured by effect size, Cramér’s V). Variables at the top of the plot have weaker links, while those with longer lines toward the right side have stronger links. Higher values suggest a bigger effect, while shorter lines mean little or no association. PSI, patient-specific instrumentation

To provide a single visual summary of the strength of these associations, a forest plot was generated demonstrating the effect size (Cramér’s V) for each demographic, surgical and anatomical variable. Glenoid retroversion and superior inclination demonstrated the strongest associations, followed by glenoid augment requirement. Variables such as age, gender, AP width, vault depth and central screw length showed minimal or no effect on PSI adoption (Figure 1).

## Discussion

This study is a decision-analysis of PSI use in shoulder arthroplasty, examining the anatomical and operative factors influencing the decision to use PSI. It does not assess clinical effectiveness, patient-reported outcomes or long-term benefits associated with PSI use.

Our study showed that PSI was used mostly for rTSA cases and was infrequently used for aTSA. Our study suggests that PSI is especially valuable and selectively adopted in cases of complex glenoid anatomy, particularly for marked retroversion, excessive superior inclination, and the need for large augments.

We noted that PSI was used in over 80% of cases with glenoid retroversion exceeding 10° and in nearly 90% of cases with superior inclination exceeding 10°. This is consistent with recent comparative studies, which support the use of PSI to improve accuracy in version and inclination, especially in technically demanding cases. Hao et al. found that surgeons using preoperative 3D planning without PSI had statistically significant deviations from planned trajectories, exceeding 10° of error when using standard instrumentation in B2 and B3 glenoids [9]. Hendel et al. also concluded that the greatest benefit of PSI was in severely retroverted glenoids (>16° retroversion): deviation averaged just 1.2° with PSI versus 10° with standard methods (P < 0.001) [10].

Additionally, there were 75 cases where PSI was not used in our study. Jacquot et al. suggested in 2018 that 3D preoperative planning alone may allow for accurate component positioning, even freehand [11]. However, PSI provides an additional margin of safety and accuracy in cases of substantial glenoid retroversion, making them particularly useful in challenging anatomical scenarios. Our study findings are concordant with those of existing literature.

Recent evidence demonstrated that PSI use in TSA is associated with a significantly lower revision rate compared to conventional instrumentation, particularly for reverse shoulder arthroplasty. Registry data have reported lower revision rates with PSI compared to conventional instrumentation, particularly for reverse shoulder arthroplasty (seven-year revision rate 4.2% vs. 5.2%), although our study did not assess clinical outcomes, and no causal conclusions can be drawn [12].

Our study demonstrated increased use of PSI with the use of augments, particularly with the use of large and medium augments. Use of augments reduces the need for eccentric reaming, minimises bone loss and better lateralisation. This allows for improved fixation and minimised substantial reaming, especially in severe deformity cases. This has been well supported in the literature. Bauer et al. demonstrated that augments help achieve a better tilt, lateralisation and better fixation options in the best-quality bone available [13]. With the advent of navigation/PSI benefits of augment placement lead to predictable correction of deformity [14]. The study also demonstrated conservation of glenoid bone by 50% with the use of augments. Our study showed a positive correlation, though not statistically significant, towards the use of a longer screw length in the PSI group.

The use of PSI guided by 3D preoperative planning has been shown not only to improve component alignment but also to enhance screw fixation strategies in the literature. Several studies demonstrate that PSI enables the placement of longer screws, which can improve baseplate fixation and initial stability. Yung et al. reported mean screw lengths exceeding 40 mm with PSI versus approximately 30 mm with conventional instrumentation [15]. Roche et al. demonstrated that using longer screws, even when fewer screws are inserted, can significantly enhance overall fixation [16]. While there was a positive correlation for use of PSI with increased glenoid deformity, the size of the glenoid did not significantly correlate with the use of PSI in our study. The findings of our study align with the existing literature. Complex angular deformities take precedence when prioritising the use of PSI. Pure dimensional values of width and depth did not serve as a driving factor for the surgeon in choosing PSI [17]. 

Strengths and limitations 

The study has certain strengths. All cases used the same preoperative CT imaging protocol and virtual planning workflow, and were performed by the same surgical team. This consistency reduces variation in PSI selection and makes the observed associations more reliable, even though causation cannot be assumed.

This was a single-centre, two-surgeon retrospective study. Certain institutional practices may have contributed to selection bias and influenced decision-making. As a retrospective analysis, the decision to use PSI may have been influenced by unmeasured factors, introducing potential decision bias. Additionally, inter-observer reliability of glenoid measurements and planning decisions was not assessed. Both surgeons were very experienced in the field, and this may have played a part in opting out of PSI in cases where a relatively less experienced surgeon might have opted for one.

We focused extensively on glenoid morphology, excluding humeral factors, which certainly play an important role in decision-making. Our study did not include the costs incurred and the time to manufacture a PSI guide, which may have a potential role in certain institutions.

## Conclusions

PSI is preferentially adopted in reverse shoulder arthroplasty cases with increased glenoid deformity and the need for medium and large augments, while demographic and simple dimensional parameters such as AP width and vault depth do not drive guide selection.

This study identifies the anatomical and operative factors that influence the use of PSI in shoulder arthroplasty. Although no decision pathway was tested, the findings describe current practice and may help guide future work on more consistent PSI selection.

Although PSI helps in improving glenoid position, the clinical and functional implications of this are yet to be debated. Further multi-centre trials may strengthen our findings and shed light on additional variables that may play a role in choosing PSI.
